# Using Distance Communication for the User-Centered Development of a Smartphone-Based Serious Game for Children With Type 1 Diabetes: Participatory Design Approach

**DOI:** 10.2196/33955

**Published:** 2022-03-29

**Authors:** Jannie Nørlev, Christina Derosche, Katrine Sondrup, Ole Hejlesen, Stine Hangaard

**Affiliations:** 1 Department of Health Science and Technology Aalborg University Aalborg Øst Denmark; 2 Steno Diabetes Center North Jutland Aalborg Denmark

**Keywords:** type 1 diabetes, children, serious game, distance communication, user-centered approach, evaluation, playtest, mobile phone

## Abstract

**Background:**

The complications of type 1 diabetes (T1D) can be delayed or prevented in children with T1D who receive proper self-management education. Smartphone-based serious games are increasingly being used as an effective tool for teaching self-management. When developing a serious game, it is important that the development process be user-centered. Traditionally, different face-to-face methods have been used when children participate in the development process. However, face-to-face data collection is not always feasible. In such situations, distance communication can be used when developing a serious game.

**Objective:**

The objective of this study is to develop a user-centered smartphone-based serious game that teaches self-management focused on carbohydrate intake in children aged 8-14 years with T1D using distance communication in both the development and evaluation of the game.

**Methods:**

The development and evaluation of a smartphone-based serious game prototype was inspired by the Lean principles, and a user-centered approach was applied. The development process included 1 expert interview and design workshops with children with T1D. On the basis of the interview and design workshop results, a serious game prototype was developed using Microsoft PowerPoint. The evaluation of the serious game prototype included an interview with a dietitian and a playtest with children with T1D. All data were collected using distance communication.

**Results:**

A user-centered smartphone-based serious game prototype was developed and evaluated. The expert interview with the dietitian formed the basis for the learning outcomes in the game. Four children and their parents contributed to the preferences, needs, requirements, and ideas for selected parts of the game design. The dietitian evaluated the prototype positively and validated its content and accuracy. The serious game prototype was well-received by the children and their parents during the playtest. The serious game prototype was perceived as a useful and engaging way to learn. However, the difficulty level was not appropriate, and the information was too basic for participants who had been diagnosed over a year ago. The use of digital communication platforms did not cause any problems.

**Conclusions:**

The smartphone-based serious game prototype has the potential to be a useful and attractive tool for teaching disease self-management. The use of distance communication proved to be a useful approach in the development of a serious game.

## Introduction

### Background

Type 1 diabetes (T1D) is one of the most common chronic diseases diagnosed in children aged <15 years [1-3]. More than half a million children are affected by T1D worldwide [3]. It is estimated that the incidence of T1D among children aged <15 years is increasing in many countries. The annual increase is estimated to be approximately 3% [3].

Children with T1D constitute a vulnerable health demographic [4]. Living with T1D is intrusive and requires a high degree of self-management to achieve glycemic control [3,5,6]. Self-management refers to tasks that an individual must undertake to control the disease and minimize the risk of short- and long-term complications [7,8]. Proper T1D self-management is complex and requires monitoring multiple daily blood glucose levels, controlling and counting carbohydrates, controlling physical activity, and managing insulin doses using multiple daily insulin injections [2,3,9,10]. Consequently, every time children with T1D play sports or eat food, they must consider how these activities will affect their blood glucose levels [2]. Self-management is further complicated by the unknown carbohydrate content of food served outside the home [11].

Owing to the complexity of self-management, children rarely manage their care alone. Parents most often play a key role and are responsible for managing T1D in children [2,11,12]. In carbohydrate counting, parents have a great responsibility, as this is particularly difficult for the child [11].

Despite help from parents, many children with T1D are unable to manage T1D or attain glycemic control [2,13]. Less than 8% of children with T1D (aged 6-12 years) and less than 7.5% of adolescents with T1D (aged 13-19 years) have optimal glycemic control [14]. Crucial factors include incomplete knowledge and understanding of treatment regimens and future health risks [10] and not following the prescribed diet [15].

Studies have indicated that it is crucial to teach children self-management and involve them in their own care from an early age to improve glycemic control [1,2,9,11,12]. Preadolescent children (aged 8-14 years) are a prime target group for developing self-management skills and understanding the effects of diet, physical activity, and insulin [14]. Teaching self-management involves facilitating and implementing the knowledge, skills, and abilities necessary for self-management [16]. In particular, knowledge of carbohydrates (eg, content and types) is essential because the amount of carbohydrates in each meal is directly related to the increase in blood glucose level and hence the dosage of insulin [17]. This knowledge can improve glycemic control, thereby reducing the risk of complications [18].

Serious games are increasingly used as tools in health education [19-22]. Serious games are digital games in which game mechanisms and characteristics are used in nongame contexts [23], such as education and health care. Children with T1D have been shown to benefit from serious games designed to teach self-management [24-26]. Serious games are effective in promoting and transferring knowledge and developing self-management skills [27-30]. These games stimulate problem-solving, reflection, and hypothesis testing [19,21,24,25]. Moreover, improvements in short- and long-term memory have been proven [31]. Serious games are an effective educational tool in T1D because they are particularly engaging and appealing [20,32], which is important because children need the motivation to learn [33].

Serious games have been developed for children with T1D to teach self-management [14,29]. However, such serious games are not available in the Danish language or adapted to Danish standards and practices for self-management education. It seems that the development of a Danish T1D serious game focusing on carbohydrates would be beneficial for teaching self-management in preadolescent children (aged 8-14 years). Recently, serious games for T1D have been dominantly developed as smartphone-based apps owing to their ubiquity [14]. A smartphone is part of everyday life for most children and adolescents [26]. In total, 95% of US teens had a smartphone in 2018 [34], whereas 93% of children aged 8-15 years in the United Kingdom owned a smartphone in 2019 [35]. In 2014, around 95% of all Danish children (aged 12-14 years) had a smartphone [36]. Therefore, when developing a new serious game, smartphones appear to be an appropriate platform.

When developing a serious game for children with T1D, it is crucial to involve children with T1D in the process to arrive at an appropriate final product [5,37-40]. When the end user participates in the development process and the findings are translated into product design, the likelihood that the indented target group will engage with the final product is increased [38-42].

Children have frequently participated in the design and development of serious games [4,11,40,43,44]. Traditionally, participation has been implemented using different face-to-face methods, including semistructured interviews, participatory design workshops, and co-design sessions [4,11,43]. However, face-to-face interaction and data collection are not always possible. During the spring of 2020, the COVID-19 pandemic changed our ability to interact in person, making face-to-face data collection impossible because of restrictions. Therefore, there is a need for alternative methods to maintain social distancing in the context of COVID-19 [45].

Distance communication can be defined as synchronous communication between geographically separated participants, enabled by devices such as a telephone or computer [46]. Distance communication has previously been used to collect and analyze research data successfully [47,48]. For instance, telephone interviews have been shown to be as productive, reliable, and efficient as face-to-face interviews [49-51]. Fox et al [47] conducted synchronous web-based focus groups with young people. They suggested that the web-based environment increased disclosure related to sensitive issues and positively impacted group dynamics and the researcher-participant relationship compared with face-to-face focus groups [47]. Furthermore, video chat using Skype (Microsoft Corp) has been demonstrated to work well as a viable, alternative data collection tool for qualitative research. The use of video also counteracts the challenges of using telephones. The video allows the facilitator and participant to use nonverbal cues and prompts (eg, smiling and nodding) [48].

### Objectives

To the best of our knowledge, distance communication has not been used to conduct user-centered workshops with children. Hence, the aim of this study is to develop a user-centered smartphone-based serious game that teaches self-management to children with T1D aged 8-14 years using distance communication in both the development and evaluation of the game. This paper describes the development process, participatory design outcomes, and evaluation of the prototype. The smartphone-based serious game prototype is focused on carbohydrates, as this has been highlighted as an essential element of self-management in T1D [17,18].

## Methods

### Development Process

A smartphone-based serious game prototype for children with T1D was developed and evaluated at Aalborg University, Denmark, from December 2019 to June 2020. The development and evaluation process was inspired by the Lean principles [41] and previously published studies [4,52-55]. Both the Lean principles and previously published studies focused on rapid prototyping, turning ideas into a prototype that is then evaluated, iterated, and refined based on feedback from the end user [4,41,52-55]. A user-centered approach was applied, where the needs and ideas of the user (children with T1D) were addressed and the perspectives of multiple stakeholders, including children with T1D, their parents, and health care professionals, were taken into account.

**Figure 1:**
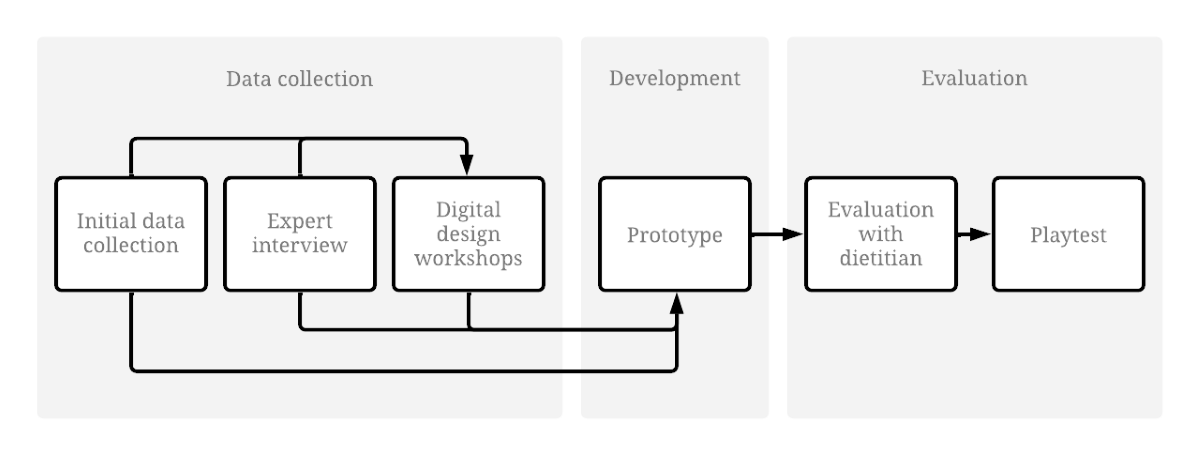
Development process for the prototype.

The development process included initial data collection, 1 expert interview, and 4 digital design workshops. Afterward, the serious game prototype was evaluated in 2 steps, including an evaluation with a dietitian and a playtest (Figure 1).

### Initial Data Collection

Before this study, a systematic scoping review was conducted to identify game mechanisms that contribute to teaching self-management. The systematic scoping review has been described in detail by Nørlev et al [29] and published elsewhere. The findings from this review were used to develop a serious game prototype. Moreover, basic information about T1D and self-management, including diet, carbohydrates [3,17,56], and serious game design and development [1,31,57-64], were collected.

### Expert Interview

An expert interview was conducted with a dietitian with >20 years of experience in educating children with T1D. The interview aimed to determine the essential knowledge about diet and carbohydrates that a child with T1D needed for self-management.

An expert interview was conducted on March 12, 2020, using FaceTime (Apple, Inc). A semistructured questionnaire consisting of open-ended questions was used (Multimedia Appendix 1). The interviews were audio-recorded and later transcribed. The transcribed data were analyzed thematically, inspired by Malterud [65]. Initial coding attached labels and themes to text segments, which appeared to indicate important information related to the research question. For each label and theme, the text segments were compiled into text that captured the essence of each theme. The transcribed materials were compared with the emerging themes. The findings were validated with participants. The first 3 authors (JN, CD, and KS) conducted the analysis.

### Digital Design Workshops

#### Overview

Four digital design workshops were conducted in April 2020. The aim was to explore the preferences, needs, requirements, and ideas for selected parts of the game design. The digital design workshop was tested in a pilot with a child aged 8 years and without T1D before data collection to determine the timeframe and intelligibility.

#### Participants

The participants (n=4; Table 1) were recruited via social media from the Danish Diabetes Association’s family network. The participants were asked to send an email to the authors if they wanted to participate. All eligible participants who responded within the deadline and met the inclusion criteria were enrolled in this study. The inclusion criteria were as follows: (1) be diagnosed with T1D; (2) be aged 8-14 years; (3) be able to speak and understand Danish or English; (4) have access to Skype, Microsoft Teams (Microsoft Corp), FaceTime, or similar app; and (5) have access to a printer. The parents were asked to be present during the digital design workshop.

**Table 1:** Participant characteristics—digital design workshop (n=4).

Participant number	Age (years)	Sex	T1D^a^ duration (years)
1	11	Male	5
2	10	Female	1
3	11	Male	3.5
4	14	Female	4

^a^T1D: type 1 diabetes.

#### Tasks and Artifacts

The digital design workshops consisted of the following three tasks: (1) suggestions for food, (2) avatars, and (3) rewards (Table 2). The tasks were based on the results of expert interviews and a systematic scoping review [29]. Visual tangible artifacts were developed for all 3 tasks (Table 2) to support cooperation by facilitating and stimulating participation, reflection, and imagination [66]. To clarify that the serious game prototype should be developed as a smartphone game, the children received a smartphone template, which they were asked to sketch on for all 3 tasks. For the second and third tasks, the participants received pictures and a description of the narrative that was decided for the game. These visual tangible artifacts were sent by email to the participants 24 hours before the digital design workshop. Participants were asked to print these artifacts.

**Table 2:** Tasks and artifacts in the digital design workshops.

Task number	Description	Artifacts	Purpose
1	Suggestions for food: sketch suggestions for food that causes blood glucose levels to rise fast and food that keep blood glucose levels stable	Smartphone template	Gain insight into relevant food to include in a game for children with T1D^a^ aged 8-14 years
2	Avatars: sketch what an avatar in a T1D game should look like	Smartphone template Description and pictures of the narrative	Obtain design ideas
3	Rewards: sketch the preferred rewards	Smartphone template Description and pictures of the narrative	Obtain design ideas

^a^T1D: type 1 diabetes.

#### Procedure

Digital design workshops were held using FaceTime. All 4 children participated once, individually, for a duration ranging from 60 minutes to 90 minutes. All 4 digital design workshops were managed by a facilitator. The facilitator introduced the first task. The child was then asked to draw the answer. When the child had finished, the facilitator asked the child to describe the drawing. The facilitator asked questions to validate and clarify the child’s thoughts and intentions with the drawing while taking notes. If the child made any comments while drawing, the facilitator also took notes. This procedure was repeated for the last 2 tasks. During the entire digital design workshop, the facilitator was able to see and communicate with the children and parents through FaceTime. The child could ask the facilitator for help at any time.

#### Data and Analysis

The digital design workshops were recorded (audio and video), transcribed by the authors afterward, and supplemented with the facilitator’s notes. The participants and their parents were asked to take a photo of their drawings from each of the 3 tasks and send them via email. This provided 3 types of data (Table 3). The photos and transcribed materials formed the core data for the analysis, whereas the notes added context and framing.

For each of the 3 tasks, the drawings, transcribed recordings, and facilitators’ notes from the digital design workshops were compared to classify the objects in the drawings. The first 3 authors (JN, CD, and KS) identified and labeled common features across the drawings, as well as unique features for each drawing individually. The findings were noted in a matrix and discussed by the authors until an agreement was reached. A summary of the findings from each of the 3 tasks was provided. Afterward, the findings were compared with transcribed recordings to validate the authors’ interpretations.

**Table 3:** Types of data from the digital design workshops.

Type	Description	Status
Photos of drawings	Photos of the drawings made during the 3 tasks	Core data
Recordings	Transcribed recordings of all 4 digital design workshops	Core data
Facilitator’s notes	Notes written down during and after each digital design workshop	Contextualizing data

### Development of the Smartphone-Based Serious Game Prototype

A smartphone-based serious game prototype was developed as a hi-fi prototype using Microsoft PowerPoint (Microsoft Corp). The serious game prototype contained effects, including animations and sounds, to make it feel like a real game. Before the development, a requirements specification was performed, as well as a plan for the user interface.

### Evaluation

The serious game prototype was evaluated in the following two steps: an evaluation by a dietitian and playtests with children with T1D.

#### Step 1: Evaluation With Dietitian

A dietitian evaluated the serious game prototype. This evaluation aimed to validate the content and accuracy of the serious game prototype, including the evidence base, health information, and learning materials. Before the evaluation, screen captures of the serious game prototype were e-mailed to the dietitian. The evaluation was conducted via phone. Each screen capture of the serious game prototype was reviewed individually, and the dietitian was asked to comment on each screen. The evaluation was audio-recorded, and notes were taken. All comments were reviewed and incorporated into the serious game prototype before the second step of the evaluation.

#### Step 2: Playtest

##### Overview

The serious game prototype was evaluated through a playtest to explore whether it engendered the experience for which it was designed. The playtest could find problems early and help confirm whether the game was suitable for the intended audience [67]. The playtest was designed to answer several key questions (Textbox 1).

**Key questions in the playtest.**

**Main question**

- Is the game engaging and appropriate for children with type 1 diabetes (T1D) aged 8-14 years?

**Supplementary questions**

- Are all 4 mini games appealing?
- Is the level of difficulty in the mini games appropriate?
- Do the children with T1D believe they learn something from the game?
- Are the children with T1D bored, confused, or frustrated when playing?
- How can the game be improved?

##### Participants

Children and parents from the digital design workshops were invited via email to evaluate the serious game prototype through a playtest. Two children and their parents participated in the study. The children were girls, aged 10 and 14 years. The serious game prototype was e-mailed to the children and parents immediately before the playtest.

##### Procedure

Before the evaluation, a protocol for the playtest was provided. The playtests were conducted using FaceTime. Both children participated once, individually. Each child had 1 parent present during the playtest. Two authors (JN and KS) conducted the playtest. The child and the parent received an oral explanation. Both the child and parent were encouraged to vocalize their thoughts and reactions while the child played the game. The child was guided to play all 4 minigames. After finishing 1 minigame, the facilitator asked follow-up questions if needed. The playtest was completed through a postgame follow-up interview with the child and parent. A script was used for the open-ended follow-up questions.

##### Data and Analysis

The playtest sessions were audio- and video-recorded and directly observed. The recordings were then transcribed. The data were analyzed and coded based on key questions. During the initial coding, labels and themes were attached to text segments that appeared to indicate important information in relation to the key questions. Labels and themes and the belonging text segments were entered into a matrix and discussed until an agreement was reached. A summary was written for each label and theme to capture the essence of each theme. The first 3 authors (JN, CD, and KS) participated in the analysis and coding.

### Ethical Considerations

Participants were invited to participate through written information on social media (digital design workshops) or email (expert interviews and evaluations). After enrollment in the study, all participants (children, parents, and dietitians) received written information. Verbal information was provided before data collection by the facilitator to the participants, who had the opportunity to ask questions. All the participants were informed that they had the right to withdraw their consent at any stage. All data collection was performed after the participants had accepted and signed an informed consent form. The parents were asked to be present during the digital design workshop and evaluation. Only authors and facilitators directly involved in data collection were present in the room during the data collection. No ethical approval was sought for this human factors study, as this was determined to be unnecessary according to guidelines outlined by the Danish National Committee on Health Research Ethics.

## Results

### Results From the Expert Interview

The expert interview mapped the essential knowledge about diet and carbohydrates that a child with T1D needed to learn to self-manage. Three educational themes emerged: carbohydrate counting, types of carbohydrates, and tips and tricks (Textbox 2). These themes were implemented in the serious game prototype.

**Themes identified from the expert interview.**

**Carbohydrate counting**

- Counting and assessing carbohydrate content in a meal
- The link between the amount of carbohydrates, blood glucose level and the required dose of insulin

**Types of carbohydrates**

- Fast and slow carbohydrates
- Food items containing fast and slow carbohydrates
- How the different types of carbohydrates affect blood glucose levels

**Tips and tricks**

- How to make carbohydrate counting easier

### Results From the Digital Design Workshops

#### Overview

The digital design workshops provided preferences, requirements, and design ideas for the selected parts of the game. The children were engaged and focused during the digital design workshops. They expressed their thoughts and elaborated on what they had drawn and why.

#### Task 1: Suggestions for Food

**Figure 2:**
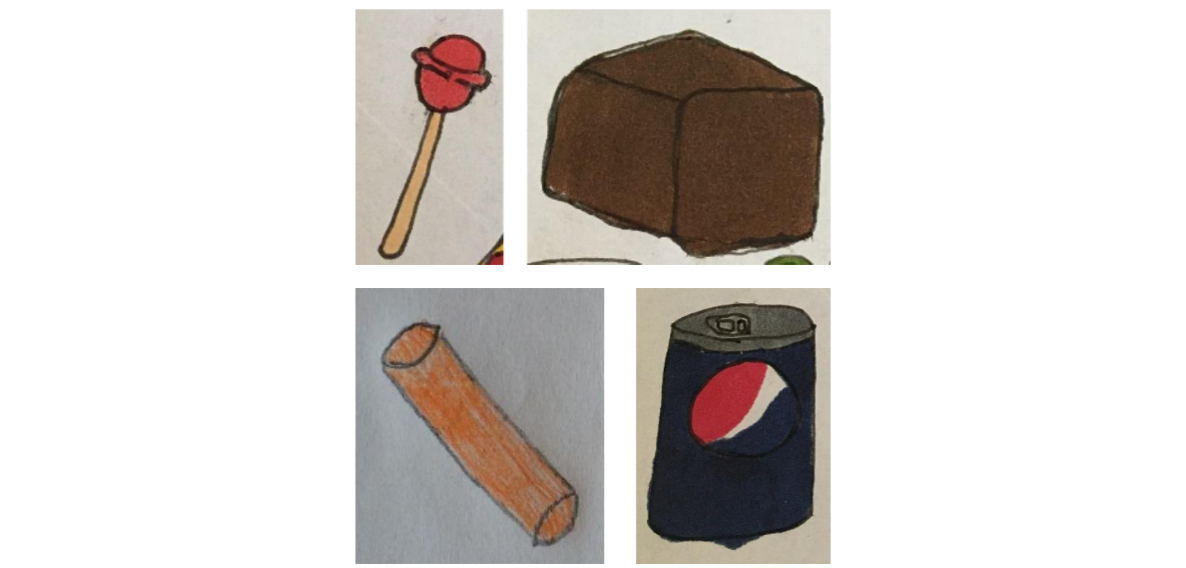
Example drawings from the digital design workshops’ task 1: suggestions of food (candy [lollipop], rye bread, carrots, and soda with sugar).

All 4 children provided several suggestions for different types of food (Figure 2; Table 4). Candy, soda with sugar, and white bread appeared in most drawings to illustrate the cause of the rapid increase in blood glucose levels. All 4 children drew rye bread or wholegrain bread as a food type that maintained stable blood glucose levels.

**Table 4:** Task 1: suggestions for food and the number of occurrences across the 4 drawings.

			Occurrences (n=4), n (%)
**Food that causes a fast increase in blood glucose levels**			
	Candy (lollipops, marshmallows, candy bars, candy floss, or wine gum)	4 (100)	
	Soda (with sugar)	3 (75)	
	White bread	3 (75)	
	Cookies	2 (50)	
	Sugary breakfast cereal	2 (50)	
	Syrup or honey	1 (25)	
	Pasta (white)	1 (25)	
**Food that keeps blood glucose levels stable**			
	Rye bread or wholegrain bread	4 (100)	
	Vegetables (carrots, cucumber, cauliflower, cabbage, or avocado)	4 (100)	
	Oats (and porridge)	2 (50)	
	Water or tea	2 (50)	
	Natural yogurt	1 (25)	
	Eggs	1 (25)	

#### Task 2: Avatars

**Figure 3:**
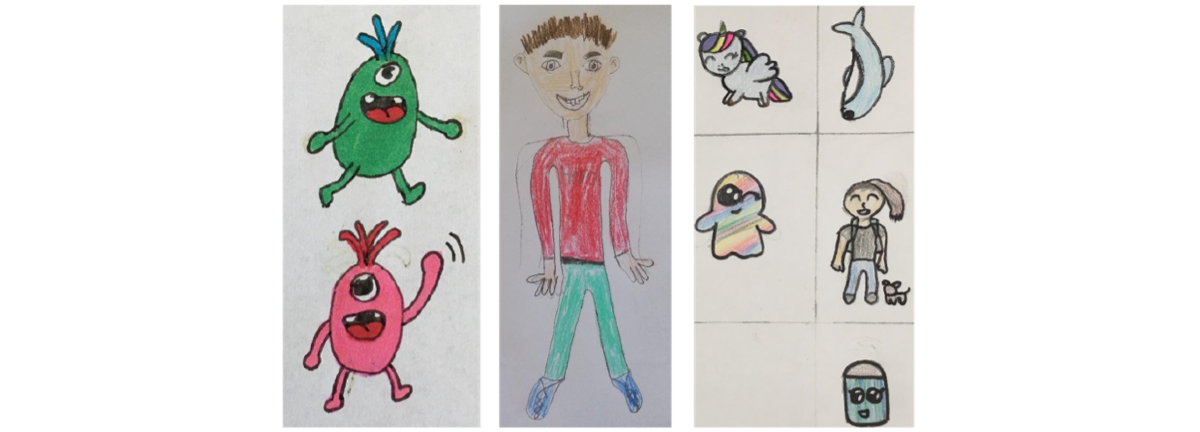
Example drawings from the digital design workshops’ task 2: suggestions for the avatars. From the left: eggs with arms and legs; a human; and different suggestions including a unicorn, dolphin, ghost, human, and cupcake.

An avatar was illustrated as a human in 3 drawings and as a cartoon character in 2 drawings. Of the 4 children, 2 (50%) preferred several avatars to choose from, whereas the other 2 (50%) children only drew 1 avatar. Of the 4 children, 3 (75%) children wanted to name the avatar themselves and 2 (50%) children appreciated being able to customize the avatar. Furthermore, all the children said that the avatar should be diagnosed with T1D and be nice (Figure 3).

#### Task 3: Rewards

**Figure 4:**
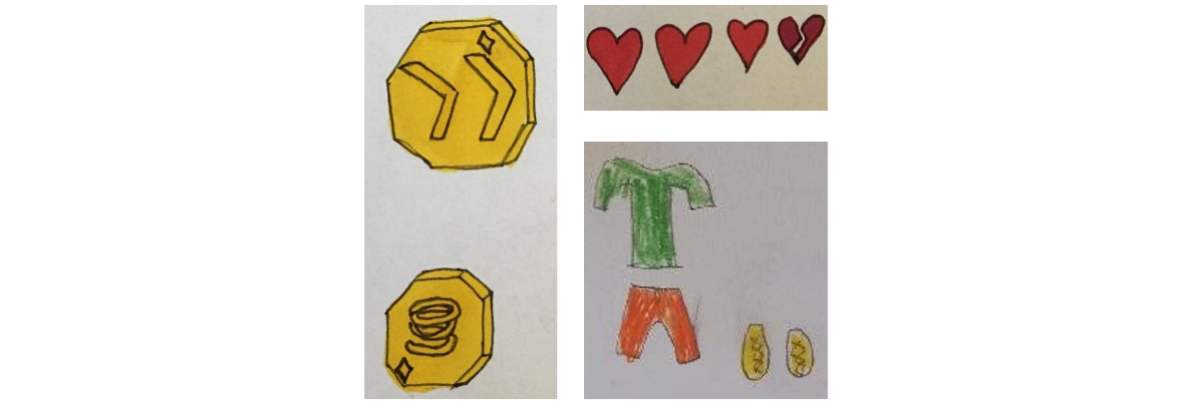
Example drawings from the digital design workshops’ task 3: suggestions for rewards (coins, extra lives, and accessories [clothes]).

Several suggestions for the types of rewards emerged in the drawings, including points, coins, superpowers, accessories for the avatar, keys and tokens, tools, and extra lives or chances (Figure 4). Of the 4 children, 2 (50%) children thought that the points should be accumulated and used to place the player on a high score list. Of the 4 children, 1 (25%) child stated that both points and coins should be used to buy things and advantages in the game. Superpowers, keys and tokens, and tools should unlock or provide different benefits to the game. According to the children, rewards should be earned when completing a level or task, when making a correct choice in the game, and according to their performance. Of the 4 children, 1 (25%) child said that rewards should be given at random intervals when playing and 1 (25%) child expressed that earned rewards should be lost when making an incorrect choice.

### Presentation of the Smartphone-Based Serious Game Prototype

The smartphone-based serious game prototype revolved around T1D self-management and specifically intended to teach children with T1D about carbohydrates and their effects. Textbox 3 lists the learning objectives and outcomes for the prototype.

The following seven game mechanisms were included in the serious game prototype: (1) narrative context, (2) feedback, (3) avatar, (4) simulation, (5) goals, (6) levels, and (7) social interactions.

**Learning objectives and outcomes.**

**Learning objectives and outcomes of the serious game prototype**

- The child should be able to differentiate between different types of carbohydrates.
- The child should understand how different types of carbohydrates affect their blood glucose level.
- The child should be able to react to high and low blood glucose levels.

When the game begins, the player must choose and name an avatar. The avatar then presents the story behind the game through speech bubbles. The avatar reports that it is diagnosed with T1D and lives inside the human body, where it must explore the impact of different types of carbohydrates in 4 different minigames. The player’s mission is to make correct decisions about carbohydrates to help the avatar control its blood glucose levels and prevent health consequences (hypoglycemia and hyperglycemia).

**Figure 5:**
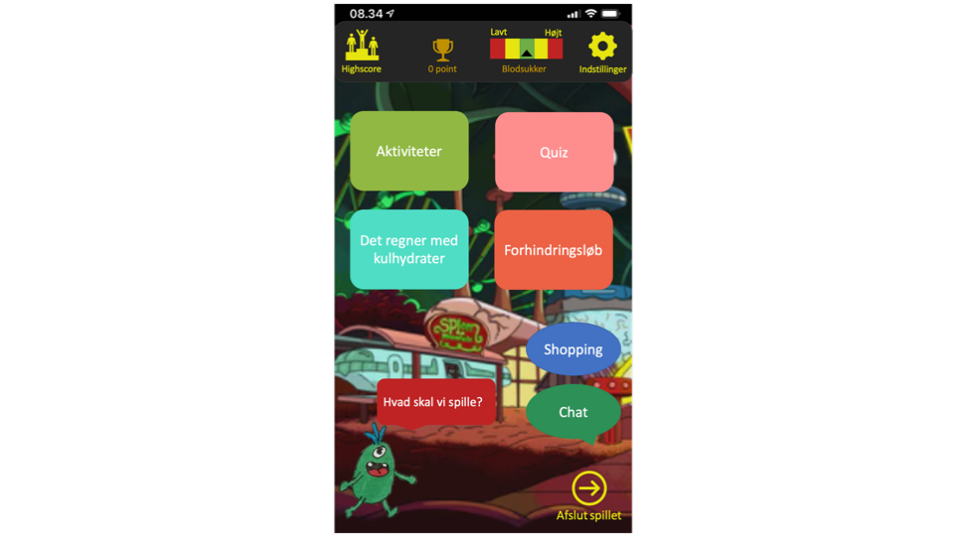
The main menu.

**Figure 6:**
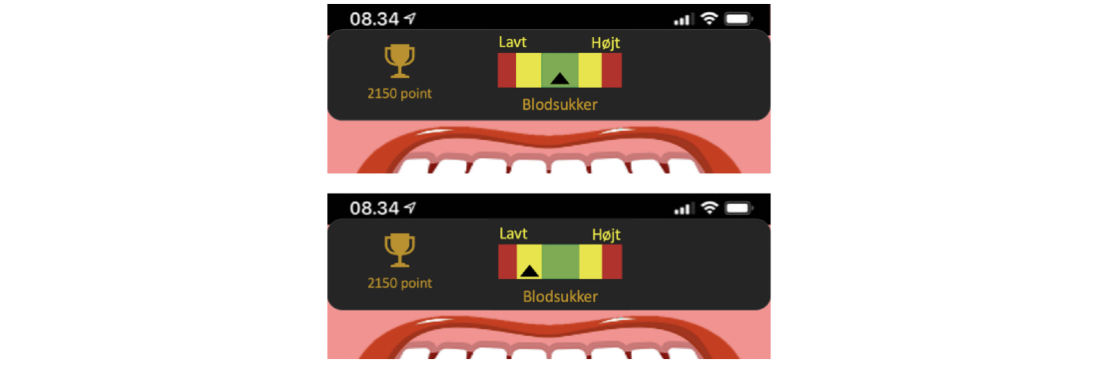
The blood glucose meter.

After familiarizing the player with the narrative context, the main menu appears (Figure 5), and the avatar demonstrates the main features. The avatar shows the blood glucose meter at the top of the screen as an imprint of its current blood glucose level throughout the 4 minigames (Figure 6). The blood glucose meter provides the player with feedback on their performance. If the player makes the correct choice, the blood glucose level will remain or return to normal (the green zone in the middle of the meter). If the player makes an incorrect choice, the blood glucose level will drop (left side of the meter) or become too high (right side of the meter), depending on the choice made. If the blood glucose level becomes too low or too high, the avatar’s facial expression changes, resulting in symptoms of hypoglycemia or hyperglycemia.

There were 4 different minigames in the serious game prototype. The first minigame is *Activities*, which is a simulation game. *Activities* teaches the player about the relationship among food (carbohydrates), blood glucose level, and physical activities and how different types of food can affect the blood glucose level after physical activities. *Activities* simulates different physical activities (eg, football) that affect the blood glucose level. The avatar points out how the player should help it choose the correct food to maintain normal blood glucose levels (green zone). The choice affects the blood glucose level and the avatar will indicate whether the choice was correct or incorrect.

The second minigame is *Quiz*, which is a quiz game. The *Quiz* aims to teach the player about the different types of carbohydrates and how they influence the blood glucose level. All questions were about carbohydrates and blood glucose levels. The avatar points out how the player must drag and drop the correct food item on the avatar to answer the question. This level contains 10 questions in total. If all 10 questions are answered correctly, a new level begins. The game ends when the player provides an incorrect answer. This level must then be repeated.

The third minigame is *It’s raining carbohydrates*, which is an arcade game. It aims to teach the player to identify foods containing different types of carbohydrates. The avatar introduces the game and instructs the player to choose one of three challenges: *fast carbohydrates*, *slow carbohydrates*, or *blood glucose level*. If the child chooses *fast carbohydrates* or *slow carbohydrates,* they must catch the type chosen (either fast or slow) when it falls in the stomach and make other types and things pass by. At the blood glucose level, the player must catch the right food or insulin based on the current blood glucose level seen on the blood glucose meter to maintain the blood glucose level within the normal zone. The player must touch the correct food and insulin before passing the stomach. The game ends when the player catches an incorrect item or when a correct item is not caught.

The fourth minigame is the *Obstacle Course*, which is an action-adventure game. The aim of the *Obstacle Course* is to teach the child about different types of carbohydrates. The player has to help the avatar through the course to complete it. Along the way, there are several obstacles to overcome, for example, avoiding villains and fast carbohydrates, which slows down the avatar. Several items help the avatar, including different medals that give the avatar superpowers to jump higher and operate faster.

In all 4 minigames, the player could win or lose points, depending on the performance. The points are accumulated and used to place the player on a high score list. All 4 minigames contain several levels. These levels become increasingly difficult and complex to complete. Throughout the serious game prototype, the avatar provides informative feedback for correct and incorrect answers.

In addition to the 4 minigames, the serious game prototype contains a shop and a chat. In the shop, the player could buy different items to customize the avatar. In the chat, the player could communicate with other players. The chat consists of context-sensitive predefined dialogues to reduce the risk of bullying and foul language. The player must touch the message they wanted to send and a speech bubble with the message appears in the chat field.

### Results From the Evaluation

#### Results From the Evaluation With the Dietitian

In general, the feedback from the dietitian was positive and mostly pertained to the inaccurate terminology and language used. However, a few evidence-based and learning material issues were apparent, including misleading or confusing food items that could be interpreted in multiple ways, potentially causing some children to provide an incorrect answer.

The dietitian made suggestions for other response options in accordance with recommendations for a T1D diet. For instance, the dietitian recommends that children with T1D always consume juice or grape sugar if they experience a drop in their blood glucose levels.

#### Results From the Playtest

The idea of playing a game to learn about self-management was well-received by the children and their parents. In general, the serious game prototype was perceived as a useful and enjoyable way to learn within the limits of what is possible with an early prototype. Laughter, excitement, and curiosity were observed among the children. Furthermore, the children and their parents thought that they were able to learn about T1D self-management from the game and felt an increase in motivation to learn. The interactivity was more motivating than in the traditional materials they had previously used. In particular, instant feedback, including the glucometer and the avatar’s symptoms, was appreciated. Both children and parents found it easier to understand how, when, and why the blood glucose level was influenced, and they found it easier to recognize high and low blood glucose symptoms.

The children and parents appreciated that the game contained different minigames, as they thought it would appeal to the children and make the game more exciting and engaging. The quiz was particularly appealing.

The evaluation showed that the level of difficulty was not appropriate and that the information was too basic. The game was too easy, and the participants knew most of the answers. Consequently, the game became boring quickly. Both children and parents found the game suitable for newly diagnosed children or children with limited knowledge. The game would benefit from more complex tasks and knowledge to become a meaningful and helpful tool for children who have been diagnosed for a longer time or those with more knowledge. It was proposed that the player should be able to choose the time of diagnosis so that the game’s level of difficulty could be adjusted accordingly. Furthermore, it could be difficult to identify food items from drawings that sometimes confused children.

The children liked that they could choose from several different avatars. The chat also earned a positive feedback. The children and their parents appreciated the social elements and liked connecting with other children with T1D. Children’s opinions on the predefined dialogue were more diverse. The oldest child felt that it was an obstacle and wanted to be able to write messages freely, whereas the youngest child liked it. Both the children and their parents liked that the serious game prototype had a multiplayer mode. The children suggested a feature in which they could invite a friend to compete in a minigame.

## Discussion

### Principal Findings

This study describes the development and evaluation of a user-centered smartphone-based serious game prototype for children with T1D using distance communication. This study found that it is possible to develop a user-centered serious game prototype using distance communication. The serious game prototype revolves around T1D self management and focuses on teaching children with T1D about carbohydrates and their effects on blood glucose levels, highlighted as an essential element of self-management [17,18]. The serious game prototype consisted of 4 minigames. The results indicate that the serious game prototype has the potential to be a useful and attractive tool to teach self-management focused on carbohydrates to newly diagnosed children with T1D that may help prevent development of T1D-related complications. Furthermore, this study implies that the developed serious game prototype does not engage children who have been diagnosed over a year ago and the results show other options to consider for this group.

For a serious game to be successful and to provide learning, players must actively use it. Enjoyment and motivation are important criteria for learning and improving one’s skills. A child who does not enjoy an activity is unlikely to engage long enough to reap any benefits [30,33]. Minigames have been successfully used to teach self-management previously [18,30,43,68-70]. The present serious game prototype allows players to engage in 4 types of minigames. The minigames were appreciated by the children with T1D and their parents because the minigames made the serious game prototype more engaging. These findings are in accordance with past research, which concluded that children aged 7-12 years are more likely to accept simpler games [71]. Minigames are particularly relevant for children as they quickly become bored [14]. However, in the evaluation, the serious game prototype was considered boring because the minigames were too basic and easy for children with T1D. It was suggested that the serious game prototype was more appropriate for newly diagnosed children or for children with limited knowledge. This suggestion is in line with Pescare et al [72], who found that a serious game mainly devoted to distinguishing complex carbohydrates from simple carbohydrates could be more effective for children who were newly diagnosed with T1D than for children who had been diagnosed for a longer period. Therefore, adding new and more complex tasks and challenging content to the serious game prototype may help improve the longevity of play and make the game meaningful and helpful for children who have been diagnosed for a longer time and have already been taught the basics of carbohydrates [73]. In addition, the ability to tailor and customize information has been shown to make serious games more relevant to the user and increase their use [74]. DeSmet et al [75] reported that games are best tailored to both sociodemographic information (eg, age and gender) and behavioral change needs (eg, knowledge and motivation). Tailored and customized information can be considered in future versions of serious games.

Several self-management games for T1D incorporate personal data, including real blood glucose measurement data [14,71]. Holtz et al [76] demonstrated that gamifying personal data is associated with improved glycemic control, quality of life, and diabetes behavior. Personal data enhances the educational effect by making the player understand how their decisions and actions affect the subsequent gameplay and heighten the player’s intrinsic motivation to play a self-management game, resulting in improved self-management [14]. Therefore, adding the child’s data may be beneficial for serious game prototypes.

The children and parents highlighted instant feedback as it was found to promote an understanding of the relationship between actions and consequences. This feedback is similar to other studies that have demonstrated that serious games containing instant feedback and simulations effectively promote self-management [30,72]. Moreover, feedback on actions in the game contributes to experimental learning [14] by allowing players to observe how their choices enhance or hinder the desired outcome [77].

Chat and social interaction were positively evaluated. Consistent with other research, children emphasized the enjoyment of social interaction [11,12,71]. Although fun is the primary reason children play games, social interaction has been reported as the second reason to play games [77]. Peer support and socializing with others in the same situation are crucial for coping with T1D [70]. In addition, studies indicate that parental involvement in a serious game is beneficial and may play a key role in positive outcomes because parents have a strong influence on their child’s behavior [78]. Past research has demonstrated that cooperation games are comparatively better than competition games for learning outcomes [33,79] and multiplayer games lead to greater enjoyment, longer engagement, and a lower dropout rate than individual games [80].

In this study, a distance communication–based, user-centered approach was applied. Children frequently participate in user-centered development and evaluation of serious games [44]. However, unlike the distance communication approach used in this study, user-centered methods traditionally consist of face-to-face interactions [4,6,11,43]. Internet and videoconference tools are increasingly popular methods of gathering qualitative data, such as focus groups or synchronous interviews by using Skype [81,82], and have become the most feasible alternative to face-to-face data collection [82]. Videoconferencing provides synchronous interaction between the researcher and participants and allows participants to be in a familiar environment, making them more comfortable [82]. The comfort of the participants is particularly important when children participate in research [81,82]. Collecting data using virtual methods makes it possible to include geographically remote participants [81]. Therefore, the number of potential participants can be increased. In this study, distance communication allowed the authors to include participants from different regions in Denmark, which probably increased the number of participants. Most children were accustomed to using a computer and engaging in video conferencing, and all participants in this study had experience with digital tools. Therefore, as expected, distance communication may not have had a significant impact on the results. This minimal impact is in line with Lo Iacono et al [48], who concluded that remote technologies such as Skype and FaceTime offer new opportunities for researchers and work well as a data collection tool.

### Study Limitations

This study has some limitations. The small sample size and limited variation within the participant pool for each stage of the serious game design process are the major limitations of this study. The participants are presumably not representative of all Danish children with T1D aged 8-14 years, and the small sample size probably prevents the transferability and generalizability of findings to wider or other contexts. Therefore, a larger sample size is preferable. The literature states that 5 users are considered sufficient to test a prototype [83]. This must be considered in the conclusion. However, the development of the serious game prototype was inspired by the Lean principles, which focus on an iterative process consisting of gathering feedback early in the process and making improvements to the prototype accordingly [41]. Therefore, the serious game prototype developed in this study is a good starting point for future versions of a serious game for newly diagnosed children with T1D. In the future, the serious game prototype must be adjusted according to the results of the playtest and tested multiple times until users are satisfied. Furthermore, a future version of the prototype must be tested to determine whether the serious game prototype is effective in teaching self-management.

### Conclusions

This study suggests that distance communication can be an approach to allow children to participate remotely in the development process of a user-centered serious game prototype. The digital design workshops helped ensure that the serious game prototype met the user’s needs and preferences. These findings indicate that the developed serious game prototype has the potential to be a useful and attractive tool for teaching self-management suitable for newly diagnosed children or children with limited knowledge. The serious game prototype was perceived as a useful and engaging way to learn about self-management within the limits of what is possible with an early prototype and small sample size. In the future, the serious game prototype must be refined according to the results of the playtest and further evaluated to determine whether it is effective in teaching self-management.

## Appendix

Multimedia Appendix 1 Interview guide for the expert interview.

## Abbreviations

T1D: type 1 diabetes
