# Supplementary material for: Using Distance Communication for the User-Centered Development of a Smartphone-Based Serious Game for Children With Type 1 Diabetes: Participatory Design Approach
Source: JMIR Serious Games. 2022 Mar 29;10(1):e33955. doi: 10.2196/33955 (PMC9006140; doi:10.2196/33955)
Supplement: Multimedia Appendix 1 [file games_v10i1e33955_app1.docx]

Supplemental file – appendix 1

# Interview guide: expert interview

| **Introduction**  The aim of this interview is to identify and clarify essential knowledge about diet and carbohydrates a child with Type 1-Diabetes (T1D) needs to obtain to learn in order to self-manage.  Results from this interview will be used in a (serious) smartphone-based game for children aged 8-14 years with T1D. The aim of the game is to teach self-management focusing around diet and carbohydrate (one of the corner stones).   - Who are we - Aim of the interview – use of results   **Anonymity**  Your name will be anonymised and you have the right to withdraw at any stage. [Hand over informed consent and go over it with participant]. Do you have any questions regarding this?   - Anonymity and informed consent - Recording - Duration   **Recording**  The interview will be audio recorded and transcribed. When the transcription is ready we would like you to read it and validate it. We can also discuss this after the interview.  **Duration**  Approximately 30 minutes  Any questions? | |
| --- | --- |
|  |  |
| Basic information about participant | - Age? - Professional background and work experience? How long have you worked with children with T1D? |
| General information about T1D-education | - What are the age of the children you teach? When in the course of treatment do you meet them? (Newly diagnosed, diagnosed for a longer tome, or?) - Can you tell me about the T1D-education?   - How do you teach?   - For how long and how many times?   - Is it the child or the whole family you teach? What is your focus? - How much do the children know already when you meet them? - What is your experience of the child’s knowledge gain? - How have you decided on what to educate the children with T1D in? |
| Essential knowledge for T1D | - Can you tell me about what you educate the child with T1D in? - What is your main focus (knowledge) when you educate? What is most important for you that the child learns? - What is the essential knowledge (about diet and carbohydrates) a child with T1D needs in order to self-manage? |
| Closing | - Do you have something to add? - Debriefing (including participant validation) |
